# Supplementary material for: The Nuclear Transporter Importin 13 Can Regulate Stress-Induced Cell Death through the Clusterin/KU70 Axis
Source: Cells. 2023 Jan 11;12(2):279. doi: 10.3390/cells12020279 (PMC9857240; doi:10.3390/cells12020279)
Supplement: Supplementary file 1 [file cells-12-00279-s001.zip › cells-2058644-supplementary.pdf]

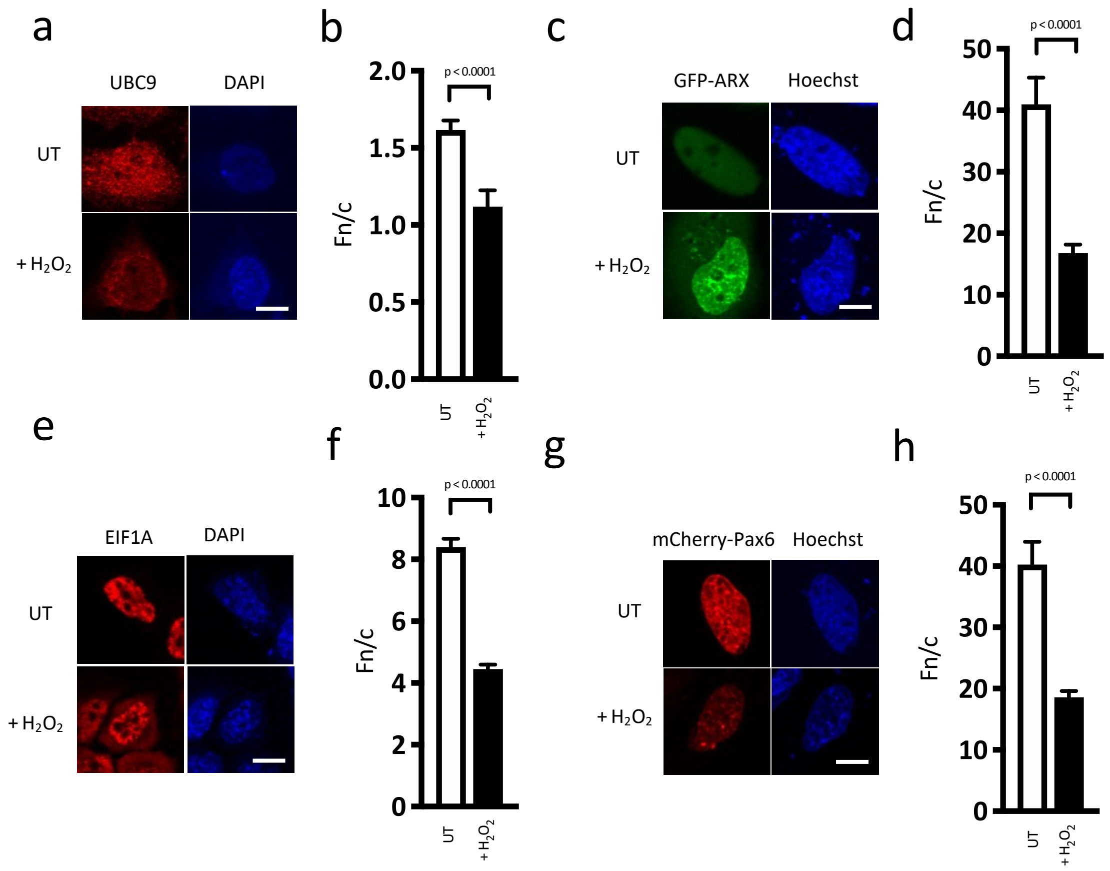

Figure S1. Analysis of IPO13 cargoes. a-b. HeLa cells treated + 125 µM H_2O_2 for 1 h prior to immunostaining with mouse-anti-UBC9 and counterstaining with DAPI were subjected to CLSM, Scale bar = 10 µM. Quantitative analysis of UBC9 localisation (**b**) was carried out using ImageJ software on images such as those in **a**, to determine the nuclear to cytoplasmic fluorescence ration (Fn/c) as described in Materials and Methods. Values represent the mean + SEM (n>25 cells) from a single typical experiment from a series of 3 similar experiments. **c-d.** Hela cells were subjected to CLSM 16 h post transfection to express GFP-ARX and treated + 125 µM H_2O_2 for 1 h prior to imaging live with a Hoechst counterstain. Quantitative analysis of GFP-ARX (**f**) was carried out as in **b**. Values represent the mean + SEM (n>36 cells) from a single typical experiment from a series of 2 similar experiments. HeLa cells were treated as in **a**. and immunostained with anti-EIF1A and counterstained with DAPI prior to CLSM. Quantitative analysis of EIF1A localisation (**f**) was carried out as in **b**. Values represent the mean + SEM (n>50 cells) from a single typical experiment from a series of 3 similar experiments. **g-h.** HeLa cells expressing mCherry-Pax6 were treated as in **c-d.** prior to Hoescht stain and CLSM imaging, Scale bar = 10 µM. **h**. Quantitative analysis of mCherry-Pax6 localisation (**h**) was carried out as in **b**. Values represent the mean + SEM (n>40 cells) from a single typical experiment from a series of 2 similar experiments.

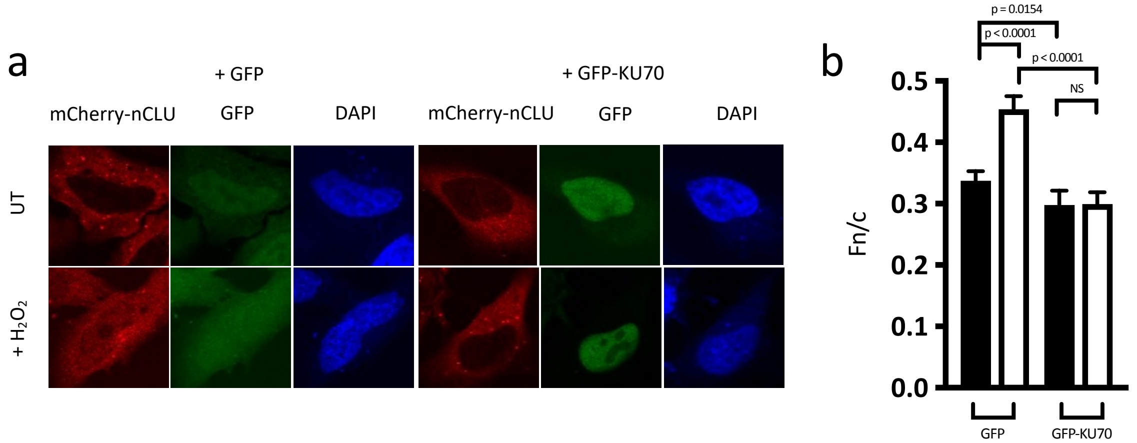

Figure S2. Stress-induced nuclear import of nCLU is impaired by ectopic expression of KU70. a. CLSM images of HeLa cells transfected to co-express mCherry-nCLU and GFP or GFP-KU70 treated 125 μM H_2O_2 for 1 h. Cells were counterstained with DAPI. Quantitative analysis of mCherry-nCLU **(b)** was carried out using the ImageJ software on images such as those in **a,** to determine the nuclear to cytoplasmic fluorescence ratio (Fn/c) of mCherry-nCLU as described in Materials and Methods, Scale bar = 10 μM. Values represent the mean ± SEM (n >40 cells) from a single typical experiment from a series of 2 similar experiments

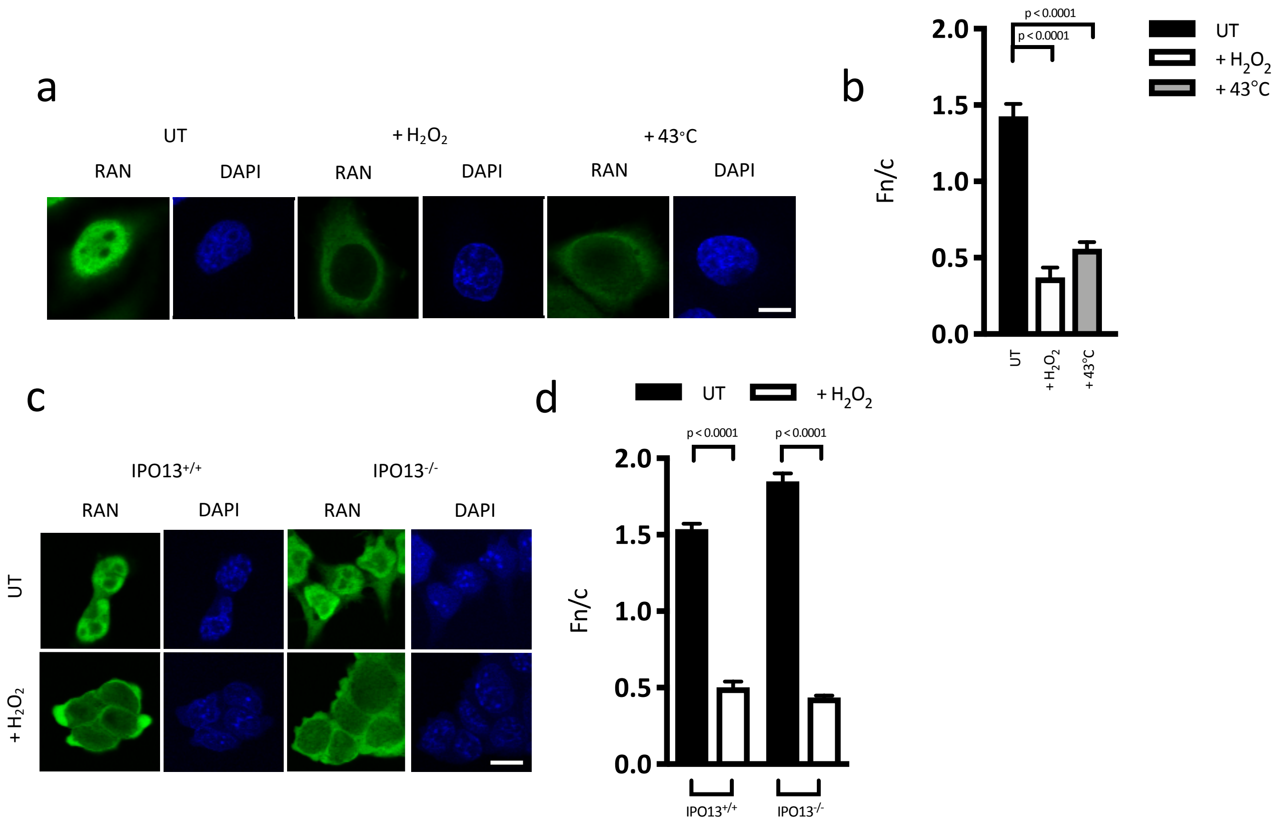

Figure S3. Stress disrupts the localisation of nuclear transport machinery in HeLa OHIO cells and mESCs. a-d. HeLa OHIO **(a,b)** and IPO13$^{+/+}$ and IPO13$^{-/-}$ ESCs **(c,d)** were treated ± 125 µM H_2O_2 for 1 h or ± 43ºC for 1 h (as indicated) prior to staining with mouse-anti-Ran (BD Biosciences) and counter staining with DAPI. Quantitative analysis of Ran **(b,d)** was carried out using the ImageJ software on images such as those in **a** and **c,** to determine the nuclear to cytoplasmic fluorescence ratio (Fn/c) of Ran as described in Materials and Methods, Scale bar = 10 µM. Values represent the mean ± SEM (n >50 cells) from a single typical experiment from a series of 2 similar experiments.

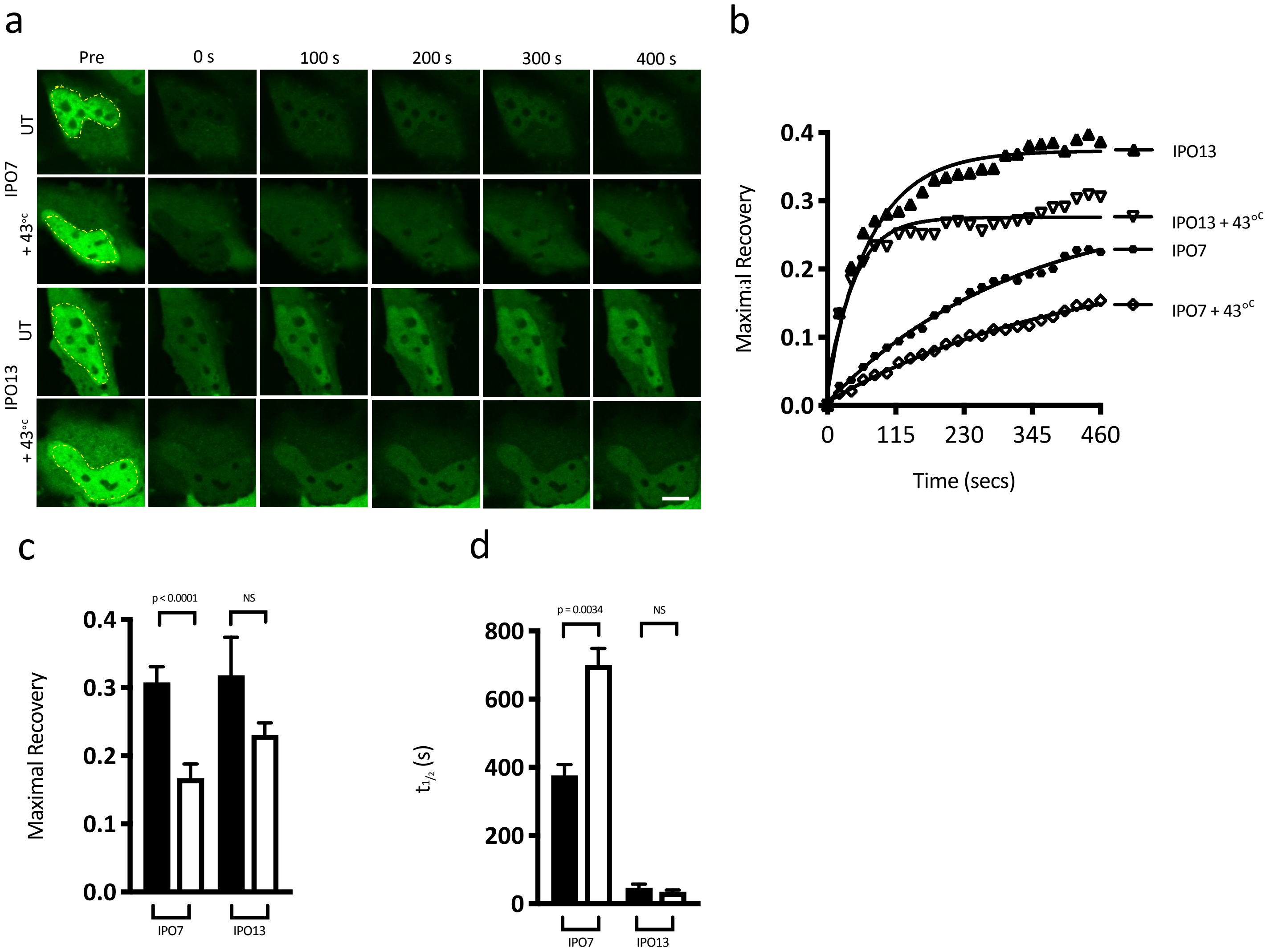

Figure S4. IPO13, unlike IPO7 continues to traffic into the nucleus under heat shock stress. a. CLSM images of HeLa cells transfected to express GFP-IPO7 or GFP-IPO13 treated ± $43^{\circ C}$ for 1 h. Cells were imaged prior to photobleaching (Pre) in the indicated nuclear region (dotted outline in yellow) and then monitored every 20 s for 8 min. **b.** Digitised images such as those in **a** were analysed to determine the fractional recovery of nuclear fluorescence (Frec(Fn-b)), Scale bar = 10 μM. Results shown are for a single representative cell under each condition. Curves such as those generated in **b**, were used to determine the maximal recovery of nuclear fluorescence (**c**), the time post-bleaching to reach half-maximal recovery ($t_{1/2}$; **d**) and the initial rate of recovery, up to 100 s, post-bleaching ((Frec (Fn-b)/s^{-1}); **e**). Results represent the mean ± SEM (n=20), typical results from 3 separate experiments. p values indicate statistical differences as determined by students t-test.

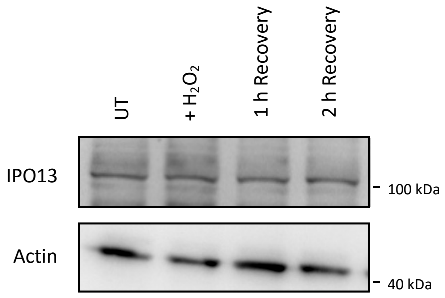

Figure S5. IPO13 expression does not change post oxidative stress. IPO13$^{+/+}$ ESCs were treated ± 125 μM H_2O_2 for 1 h, after which they were lysed either immediately or 1 or 2 h post removal of treatment for western blot analysis. Blots were probed with anti-IPO13 (Protein Tech) or anti-actin (Abcam) antibodies.

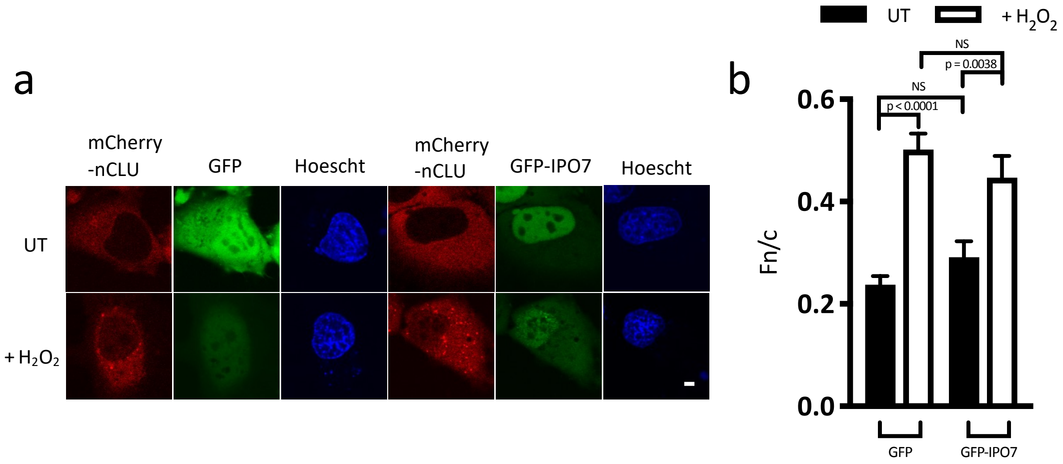

Figure S6. IPO7 overexpression does not affect nCLU localisation. a-b. Hela cells were subjected to CLSM 16 h post transfection to co-express mCherry-nCLU and GFP or GFP-IPO7 and treated $\pm$ 125 µM H_2O_2 for 1 h prior to imaging live with a Hoechst counterstain. Quantitative analysis of mCherry-nCLU (**b**) was carried out using ImageJ software on images such as those in **a**, to determine the nuclear to cytoplasmic fluorescence ration (Fn/c) as described in Materials and Methods. Values represent the mean $\pm$ SEM (n>27 cells) from a single typical experiment from a series of 3 similar experiments.
